# Partial Versus Complete Bacillus Calmette-Guérin Intravesical Therapy and Bladder Cancer Outcomes in High-risk Non–muscle-invasive Bladder Cancer: Is NIMBUS the Full Story?

**DOI:** 10.1016/j.euros.2021.01.009

**Published:** 2021-02-16

**Authors:** Michael E. Rezaee, A. Aziz Ould Ismail, Chiamaka L. Okorie, John D. Seigne, Kristine E. Lynch, Florian R. Schroeck

**Affiliations:** a White River Junction VA Medical Center, White River Junction, VT, USA; b Section of Urology, Dartmouth Hitchcock Medical Center, Lebanon, NH, USA; c Geisel School of Medicine at Dartmouth College, Lebanon, NH, USA; d Norris Cotton Cancer Center, Dartmouth Hitchcock Medical Center, Lebanon, NH, USA; e VA Salt Lake City Health Care System and University of Utah, Salt Lake City, UT, USA; f The Dartmouth Institute for Health Policy and Clinical Practice, Geisel School of Medicine at Dartmouth College, Lebanon, NH, USA

**Keywords:** Bladder cancer, Bacillus Calmette-Guérin, Immunotherapy, Transurethral resection, Outcomes

## Abstract

**Background:**

It is important to understand the implications of reduced bacillus Calmette-Guérin (BCG) treatment intensity, given global shortages and early termination of the NIMBUS trial.

**Objective:**

To assess the association of partial versus complete BCG induction with outcomes.

**Design, setting, and participants:**

This is a retrospective cohort study of veterans diagnosed with high-risk non–muscle-invasive bladder cancer (NMIBC; high grade [HG] Ta, T1, or carcinoma in situ) between 2005 and 2011 with follow-up through 2014.

**Intervention:**

Patients were categorized into partial versus complete BCG induction (one to five vs five or more instillations). Partial BCG induction subgroups were defined for comparison with the NIMBUS trial.

**Outcome measurements and statistical analysis:**

Propensity score–adjusted regression models were used to assess the association of partial BCG induction with risk of recurrence and bladder cancer death.

**Results and limitations:**

Among 540 patients, 114 (21.1%) underwent partial BCG induction. Partial versus complete BCG induction was not significantly associated with the risk of recurrence in HG Ta (cumulative incidence [CIn] 46.6% vs 53.9% at 5 yr, *p* =  0.38) or T1 (CIn 47.1% vs 56.7 at 5 yr, *p* = 0.19) disease. Similarly, we found no increased risk of bladder cancer death (HG Ta: CIn 4.7%7vs 5.4% at 5 yr, *p* = 0.87; T1: CIn 10.0% vs 11.4% at 5 yr, *p* =  0.77). NIMBUS-like induction was associated with an increased risk of recurrence in patients with HG Ta disease, although not statistically significant. Unmeasured confounding is a limitation.

**Conclusions:**

Cancer outcomes were similar among high-risk NMIBC patients who underwent partial versus complete BCG induction, suggesting that future research is needed to determine how to optimize BCG delivery for the greatest number of patients, especially during global shortages.

**Patient summary:**

Outcomes were similar between patients receiving partial and complete courses of bacillus Calmette-Guérin (BCG) therapy. Future research is needed to determine how to best deliver BCG to the greatest number of patients, particularly during medication shortages.

## Take Home Message

Patients with high-risk non–muscle-invasive bladder cancer who underwent partial bacillus Calmette-Guérin (BCG) induction experienced similar outcomes to those who received complete BCG induction. Additional prospective trials are needed to determine how to maximize BCG utilization for the greatest number of patients during global shortages.

## 1 Introduction

Non–muscle-invasive bladder cancer (NMIBC) accounts for ∼75% of bladder cancer diagnoses [1]. Using tumor characteristics, patients with NMIBC can be risk stratified based on the probability of disease recurrence and progression to muscle-invasive disease [2]. High-risk NMIBC includes high-grade (HG) Ta, T1, and carcinoma in situ (CIS). These tumors have up to an 80% risk of recurrence and up to a 50% risk of progression to muscle-invasive disease at 5 yr [3], [4].

Intravesical bacillus Calmette-Guérin (BCG) reduces tumor recurrence and progression to muscle-invasive disease [5], [6]. Guidelines recommend a 6-wk induction course of adjuvant BCG followed by maintenance for 3 yr for patients with BCG-responsive disease [2], [7]. Unfortunately, repetitive global shortages have disrupted guideline-recommended BCG therapy for many high-risk patients [8], [9]. BCG instillations have been rationed and diluted in an attempt to treat the greatest number of patients [8], [9]. However, the European Association of Urology (EAU) Research Foundation’s NIMBUS trial indicates that reducing BCG induction to three doses at 1, 2, and 6 wk is inferior to standard 6-wk induction [10].

Given these recent developments, we leveraged clinical, pharmacy, and administrative data from the Department of Veterans Affairs (VA) to assess the association of partial versus complete BCG therapy with outcomes in patients with high-risk NMIBC. Granular pharmacy data allowed not only for quantifying the number of BCG instillations given during induction, but also for varying administration schedules, for example, with no more than 2-wk pauses versus longer gaps. Understanding the effects of partial BCG treatment has important implications for high-risk patients in the setting of repetitive global shortages.

## 2 Patients and methods

### 2.1 Study population

We performed a retrospective cohort study of VA patients aged >65 yr, diagnosed with high-risk NMIBC by EAU guideline criteria (ie, HG Ta, T1, or CIS) between 2005 and 2011 with follow-up through 2014 [7]. As previously described, a validated algorithm was used to identify 2152 patients with newly diagnosed high-risk urothelial cell carcinoma of the bladder [11], [12]. Patients were excluded if they did not receive at least one instillation of BCG within the prespecified induction or maintenance windows (*n* = 1534); they were missing comorbidity (*n* = 39), transurethral resection (*n* = 34), or demographic (*n* = 4) data; or their follow-up ended during the BCG induction window (*n* = 2). The final sample consisted of 540 patients.

### 2.2 Defining partial versus complete BCG therapy

The International Bladder Cancer Group defined adequate BCG therapy as receiving (1) at least five of six BCG induction instillations and (2) at least two of three BCG maintenance instillations in a 6-mo period [13]. We characterized BCG administration in a similar fashion.

**Fig. 1:**
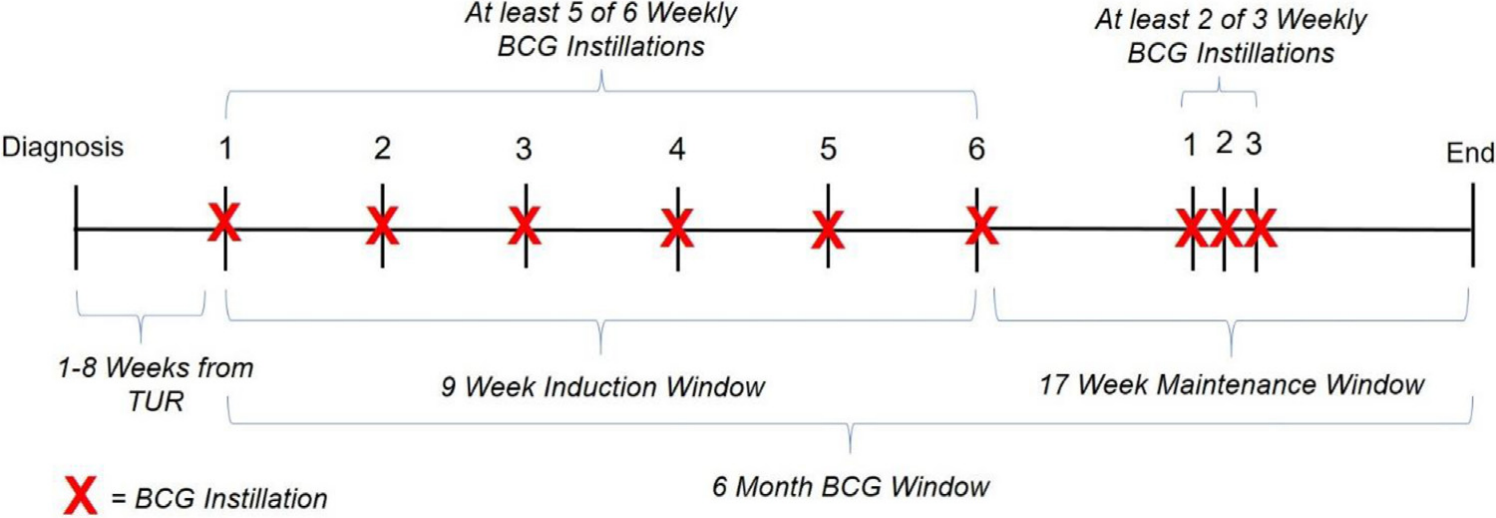
We characterized BCG administration similar to the International Bladder Cancer Group definitions for adequate BCG therapy: complete BCG induction = five or more intravesical instillations over a 9-wk induction window after first BCG administration following index transurethral resection (TUR); partial BCG induction = fewer than five BCG instillations over the 9-wk induction window. A 9-wk induction window was selected to account for potential delays in weekly intravesical therapy. Patients who underwent complete BCG induction were further categorized into those who underwent versus those who did not undergo BCG maintenance (2- or 3-weekly maintenance BCG instillations of zero to one). BCG = bacillus Calmette-Guérin; TUR = transurethral resection.

Complete BCG induction was defined as five or more instillations over a 9-wk induction window after first BCG administration following index transurethral resection (TUR; Fig. 1). Partial BCG induction was defined as receiving less than that. A 9-wk induction window was selected to account for potential delays in weekly intravesical therapy (eg, due to urinary tract infection and gross hematuria) [7]. TURs were identified using International Classification of Diseases (ICD), ninth revision (ICD-9), and Current Procedural Terminology (CPT) codes as previously described [11]. We used administrative data (Healthcare Common Procedure Coding System Code J9031) as well as VA pharmacy and immunization data to enumerate intravesical BCG instillations.

After categorizing patients by BCG induction type, patients who underwent complete BCG induction were further categorized into those who underwent versus those who did not undergo BCG maintenance (two or three maintenance BCG instillations vs zero to one; Fig. 1).

### 2.3 Outcomes

Outcomes included disease recurrence, bladder cancer death, and progression to invasive disease. Disease recurrence was defined as the first TUR with cancer in the specimen at least 90 d after the diagnosis date. TURs with cancer in the specimen were identified using ICD-9 or CPT procedure codes and validated natural language processing (NLP) algorithms of VA pathology data [12]. Date of death was obtained from the VA Corporate Data Warehouse (CDW) Vital Status File and cause of death from the National Death Index (NDI) [14]. Progression to invasive disease was defined as invasion into the lamina propria (T1) or muscularis propria (T2), and could be assessed only among patients diagnosed with HG Ta disease, due to limitations of the NLP algorithms to differentiate invasion into the lamina propria versus muscularis propria [12].

### 2.4 Statistical analysis

We compared patient characteristics by receipt of partial versus complete BCG induction therapy using descriptive statistics. A series of propensity score–adjusted analyses were then performed to assess the association between partial versus complete BCG induction and outcomes. Propensity scores were calculated using multivariable logistic regression to model each patient’s probability of undergoing partial BCG induction, conditional on the characteristics displayed in Table 1 as well as the Nosos-p score [15], [16]. The Nosos-p score is a standardized VA risk-adjustment score [17].

**Table 1:** Patient characteristics by partial and complete induction

Category	Overall (*n* = 540)	Partial induction BCG (*n* = 114)	Complete induction BCG (*n* = 426)	*p* value a
Age (yr), mean (SD)	76 (6.6)	77 (6.4)	76 (6.7)	0.17
Male sex, b*N* (%)	>529 (>98.0)	>103 (>90.3)	>415 (97.4)	0.85
Race, *N* (%)				0.71
White	455 (84.3)	97 (85.1)	358 (84)	
Black b	30 (5.6)	<11	24 (5.6)	
Asian b	<11	<11	<11	
Hispanic b	<11	<11	<11	
Native American b	<11	<11	<11	
Unknown b	40 (7.4)	<11	>30 (>7.0)	
Comorbidity, *N* (%)				0.19
0	80 (14.8)	21 (18.4)	59 (13.8)	
1	143 (26.5)	33 (28.9)	110 (25.8)	
2	129 (23.9)	19 (16.7)	110 (25.8)	
≥3	188 (34.8)	41 (36)	147 (34.5)	
Year of diagnosis, *N* (%)				0.81
2005 b	12 (2.2)	<11	<11	
2006	77 (14.3)	20 (17.5)	57 (13.4)	
2007	78 (14.4)	16 (14)	62 (14.6)	
2008	90 (16.7)	17 (14.9)	73 (17.1)	
2009	88 (16.3)	15 (13.2)	73 (17.1)	
2010	104 (19.3)	25 (21.9)	79 (18.5)	
2011	91 (16.9)	<11	<11	
Proportion living in ZIP code with ≥25% college graduates, *N* (%)	244 (45.2)	48 (42.1)	196 (46)	0.46
Living in urban vs rural area, *N* (%)				0.75
Urban	315 (58.3)	65 (57)	250 (58.7)	
Stage, *N* (%)				0.81
Ta (high grade or with CIS)	210 (38.9)	43 (37.7)	167 (39.2)	
T1	290 (53.7)	>58 (>52.0)	229 (53.8)	
Carcinoma in situ only b	40 (7.4)	<11	>28 (>5)	
Carcinoma in situ, *N* (%)	159 (29.4)	159 (29.8)	125 (29.3)	0.92
Bladder cancer grade, *N* (%)				0.41
High	502 (93)	>103 (>90.4)	398 (93.4)	
Low (all T1 tumors) b	38 (7)	<11	28 (6.6)	

BCG = bacillus Calmette-Guérin; CIS = carcinoma in situ; IQR = interquartile range; SD = standard deviation.

a From chi-square test for categorical variable and Wilcoxon test for continuous variables, the median and IQR of which were presented. Missing observations were excluded from analysis.

b Exact numbers not shown to protect confidentiality if *n* < 11.

We then used propensity score–adjusted Fine-Gray competing risk regression to evaluate the association of partial BCG induction with outcomes. Death was modeled as a competing risk in the models assessing recurrence, and death from causes other than bladder cancer was modeled as a competing risk in the models assessing bladder cancer death and progression. For each model, we calculated E-values, which indicate the strength with which any unmeasured confounder would have to be associated with both partial BCG induction and cancer outcomes for us to miss any true increase in risk by 10% associated with partial BCG induction [18]. We then performed similar analyses comparing patients who received and those who did not receive BCG maintenance to assess the association of BCG maintenance status on study outcomes (additional methods in the Supplementary material).

**Fig. 2:**
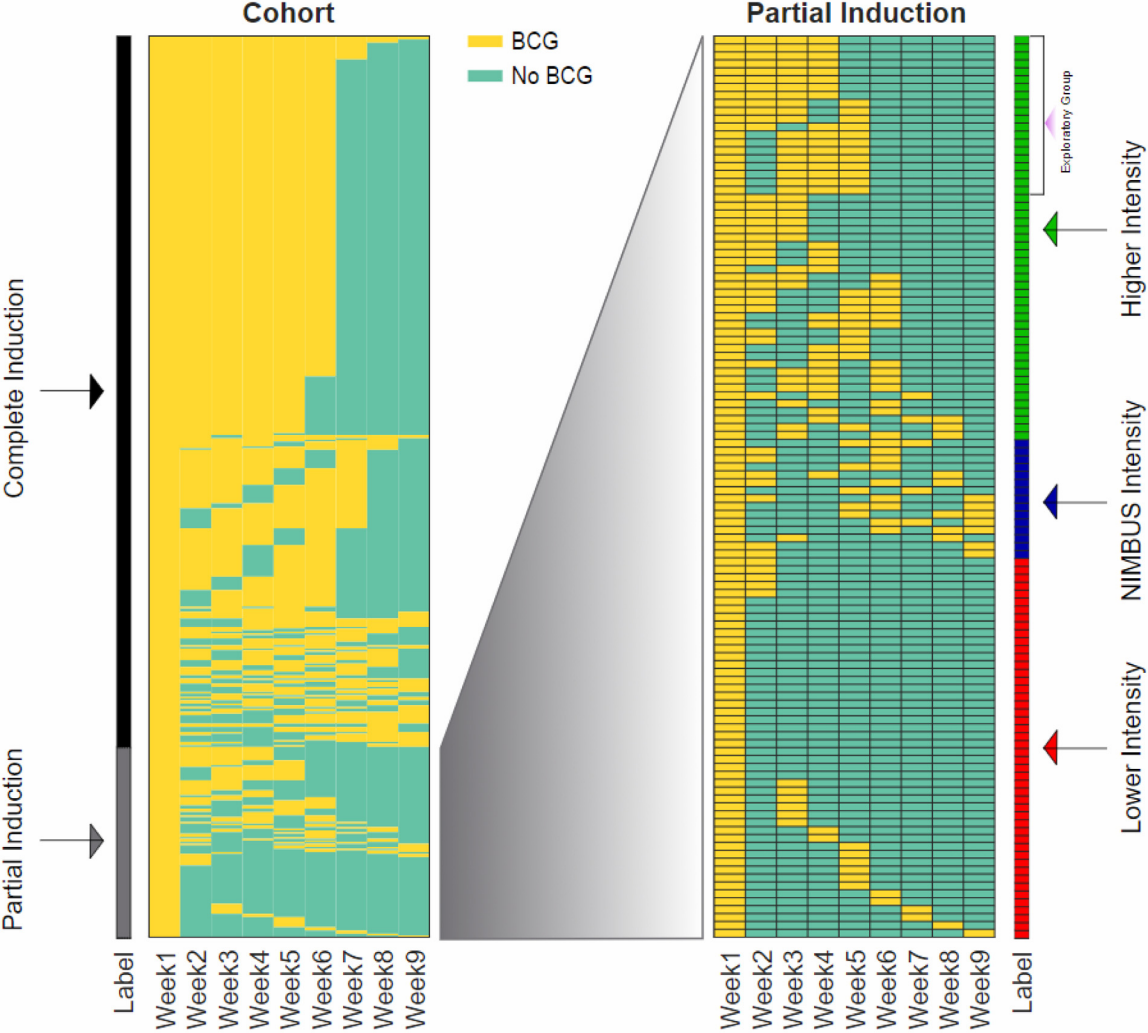
Details of BCG administration for the entire cohort (left) and the partial induction groups (right). Each row represents a patient in the cohort. For each patient, weeks with BCG administration are shown in yellow and weeks without BCG in green. Side bars indicate BCG induction groups. Higher-intensity partial BCG = three or four instillations with a ≤2-wk gap between doses; NIMBUS intensity = three or four instillations with a ≥3-wk gap between consecutive doses; and lower-intensity partial BCG = one or two instillations in the induction window with various weekly gaps between doses. BCG = bacillus Calmette-Guérin.

We examined different patterns of partial BCG induction to compare and contrast our findings with the recently halted NIMBUS trial, which examined the effect of three (at 1, 2, and 6 wk) versus six BCG instillations in the induction period [10]. We defined three partial BCG subgroups based on the number of BCG induction instillations as well as the number of consecutive weekly gaps (or “skipped weeks”) between BCG doses: higher-intensity (three or four instillations with a ≤2 wk consecutive gap), NIMBUS-intensity (three or four instillations with a ≥3 wk consecutive gap), and lower-intensity (one or two BCG instillations) partial BCG (Fig. 2). Similar Fine-Gray regression models were used to examine study outcomes by partial BCG subgroups with complete BCG as the reference. Lastly, we compared an exploratory subgroup of patients within the higher-intensity BCG subgroup with patients who received complete BCG, in an attempt to identify a more favorable partial BCG regimen. The patients in the exploratory subgroup received 4 wk of BCG, with no more than a 1 wk gap between BCG instillations.

Analyses were performed between September 2019 and December 2020. A *p* value of <0.05 was used for statistical significance. The study was approved by the Veteran’s Institutional Review Board of Northern New England (#897920) and the University of Utah Institutional Review Board (#00079402). Analyses were performed using MATLAB R2017B (Mathworks), SAS Enterprise Guide 7.15, and Stata v15.1.

## 3 Results

### 3.1 Partial versus complete BCG induction and outcomes

Of the 540 patients diagnosed with high-risk NMIBC who received at least one dose of BCG, 114 (21.1%) underwent partial BCG induction. Patients who underwent partial versus complete BCG induction received substantially fewer BCG instillations (average 2.8 vs 6.0). Patient characteristics did not differ significantly by BCG induction status (Table 1).

**Fig. 3:**
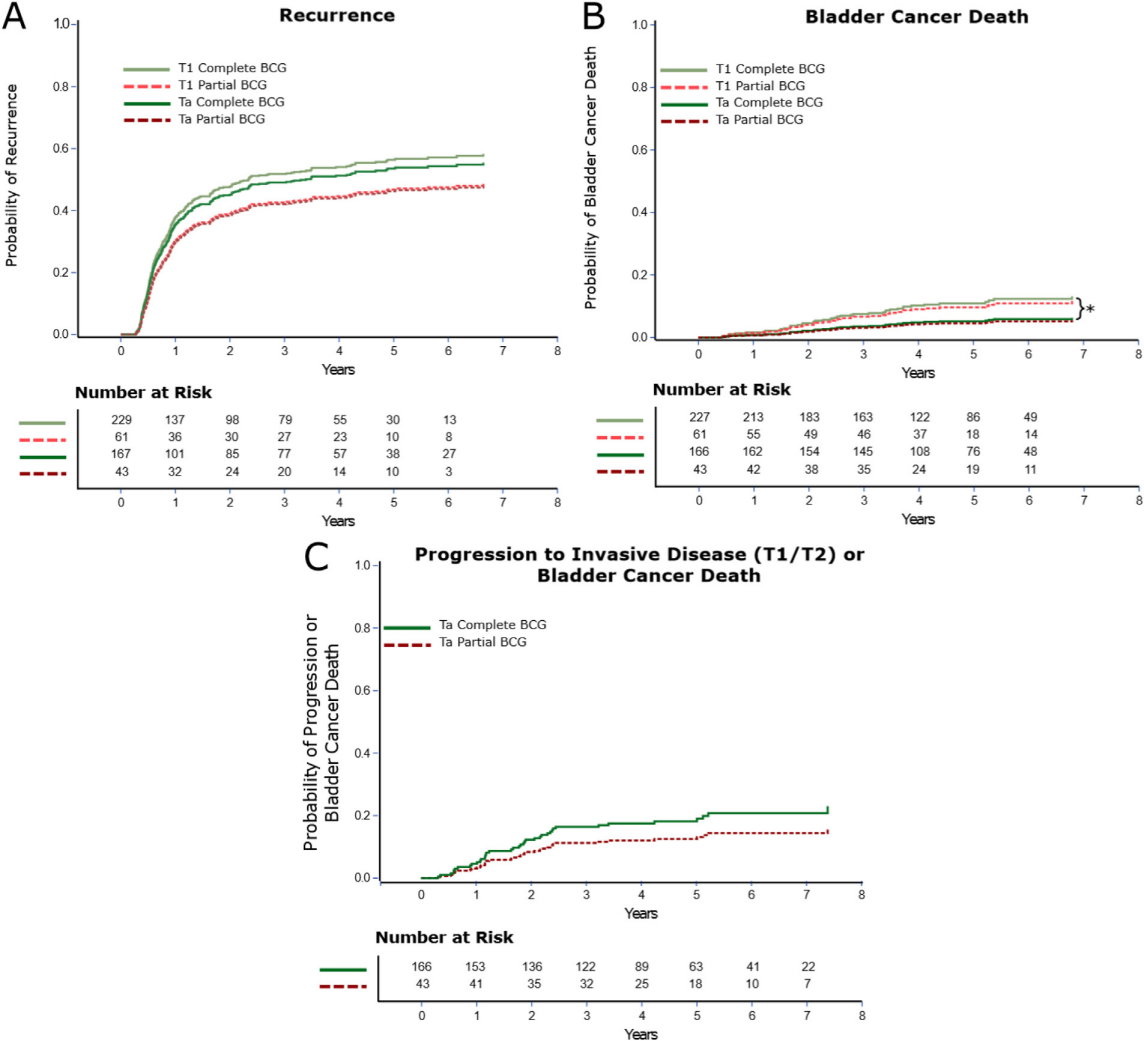
Cumulative incidence plots showing the probability of (A) disease recurrence and (B) bladder cancer death by HG Ta versus T1 disease and by BCG induction status. The bracket in Figure 3B indicates a significant difference in the risk of bladder cancer death between T1 and Ta patients who underwent complete BCG. (C) The probability of progression to invasive disease (T1 or T2) in patients diagnosed with HG Ta by BCG induction status. Data are from Fine and Gray competing risk models adjusted for propensity score, and none of the differences were statistically significant. BCG = bacillus Calmette-Guérin; HG = high grade.

At a median follow-up of 4.4 and 4.5 yr, partial versus complete BCG induction was not significantly associated with an increased risk of disease recurrence for patients diagnosed with HG Ta (cumulative incidence [CIn] 46.6% vs 53.9% at 5 yr, *p* =  0.38) or T1 (CIn 47.1% vs 56.7% at 5 yr, *p* =  0.19) disease (Fig. 3A and Supplementary Tables 2–10 for model output). Similarly, partial versus complete BCG induction was not significantly associated with an increased risk of bladder cancer death for patients diagnosed with HG Ta (CIn 4.7%7vs 5.4% at 5 yr, *p* =  0.87) or T1 (CIn 10.0% vs 11.4% at 5 yr, *p* =  0.77) disease (Fig. 3B). Among patients who received partial BCG, there was no significantly increased risk of progression to invasive disease or bladder cancer death when diagnosed with HG Ta (CIn 13.1% vs 19.0% at 5 yr, *p* =  0.37; Fig. 3C). E-values were 1.78, 1.62, and 2.17 for recurrence, bladder cancer death, and progression among Ta patients, respectively. They indicate the minimum strength of association on the risk ratio scale that any unmeasured confounder would need to have with both partial BCG induction and outcomes for us to miss a true 10% increase in recurrence, bladder cancer death, or progression.

Study outcomes did not differ significantly by receipt of BCG maintenance, although point estimates suggested worse outcomes among those who did not receive BCG maintenance (Supplementary material).

### 3.2 Partial BCG induction subgroups and outcomes

Among the 114 patients who underwent partial BCG induction, 51 (44.7%) had higher-intensity induction, 15 (13.2%) had NIMBUS intensity, and 48 (42.1%) had lower-intensity induction (Fig. 2 and Table 2).

**Table 2:** Patient characteristics by complete BCG and higher-, NIMBUS-, and lower-intensity partial BCG

Category	Complete induction BCG (*n* = 426)	Higher intensity (*n* = 51)	NIMBUS intensity (*n* = 15)	Lower intensity (*n* = 48)	*p* value a
Age (yr), mean (SD)	76 (6.7)	77 (6.4)	77 (5.1)	77 (6.9)	0.23
Male sex, b*N* (%)	>415 (>97)	> 40 (> 78)	>4 (>27)	>37 (>77)	0.05
Race, *N* (%)					0.7
White	358 (84)	> 40 (86.3)	13 (86.7)	40 (83.3)	
Black b	24 (5.6)	<11	<11	<11	
Asian b	<11	<11	<11	<11	
Hispanic b	<11	<11	<11	<11	
Native American b	<11	<11	<11	<11	
Unknown b	32 (7.5)	<11	<11	<11	
Comorbidity, *N* (%)					0.4
0	59 (13.8)	11 (21.6)	<11	<11	
1	110 (25.8)	11 (21.6)	<11	18 (37.5)	
2	110 (25.8)	<11	<11	<11	
≥3	147 (34.5)	>18 (>35.3)	<11	15 (31.2)	
Year of diagnosis, *N* (%)					0.8
2005 b	<11	<11	<11	<11	
2006	57 (13.4)	11 (21.6)	<11	<11	
2007	62 (14.6)	<11	<11	<11	
2008	73 (17.1)	<11	<11	<11	
2009	73 (17.1)	<11	<11	<11	
2010	79 (18.5)	13 (25.5)	<11	<11	
2011	<11	<11	<11	<11	
Proportion living in ZIP code with ≥25% college graduates, *N* (%)	196 (46)	24 (47.1)	<11	17 (35.4)	0.6
Living in urban vs rural area, *N* (%)					0.9
Urban	250 (58.7)	30 (58.8)	<11	26 (54.2)	
Stage, *N* (%) b					0.06
Ta (high grade or with CIS)	167 (39.2)	23 (45.1)	<s11	17 (35.4)	
T1	229 (53.8)	26 (51)	12 (80)	23 (47.9)	
Carcinoma in situ only	30 (7)	<11	<11	<11	
Carcinoma in situ, *N* (%)	125 (29.3)	15 (29.4)	<11	17 (35.4)	0.4
Bladder cancer grade, *N* (%) b					0.2
High	398 (93.4)	>40 (>78.4)	>4 (>26.7)	>37 (>77.1)	
Low (all T1 tumors)	28 (6.6)	<11	<11	<11	

BCG = bacillus Calmette-Guérin; CIS = carcinoma in situ; IQR = interquartile range; SD = standard deviation.

a From chi-square test for categorical variable and Wilcoxon test for continuous variables, the median and IQR of which were presented. Missing observations were excluded from analysis.

b Exact numbers not shown to protect confidentiality if *n* < 11.

**Fig. 4:**
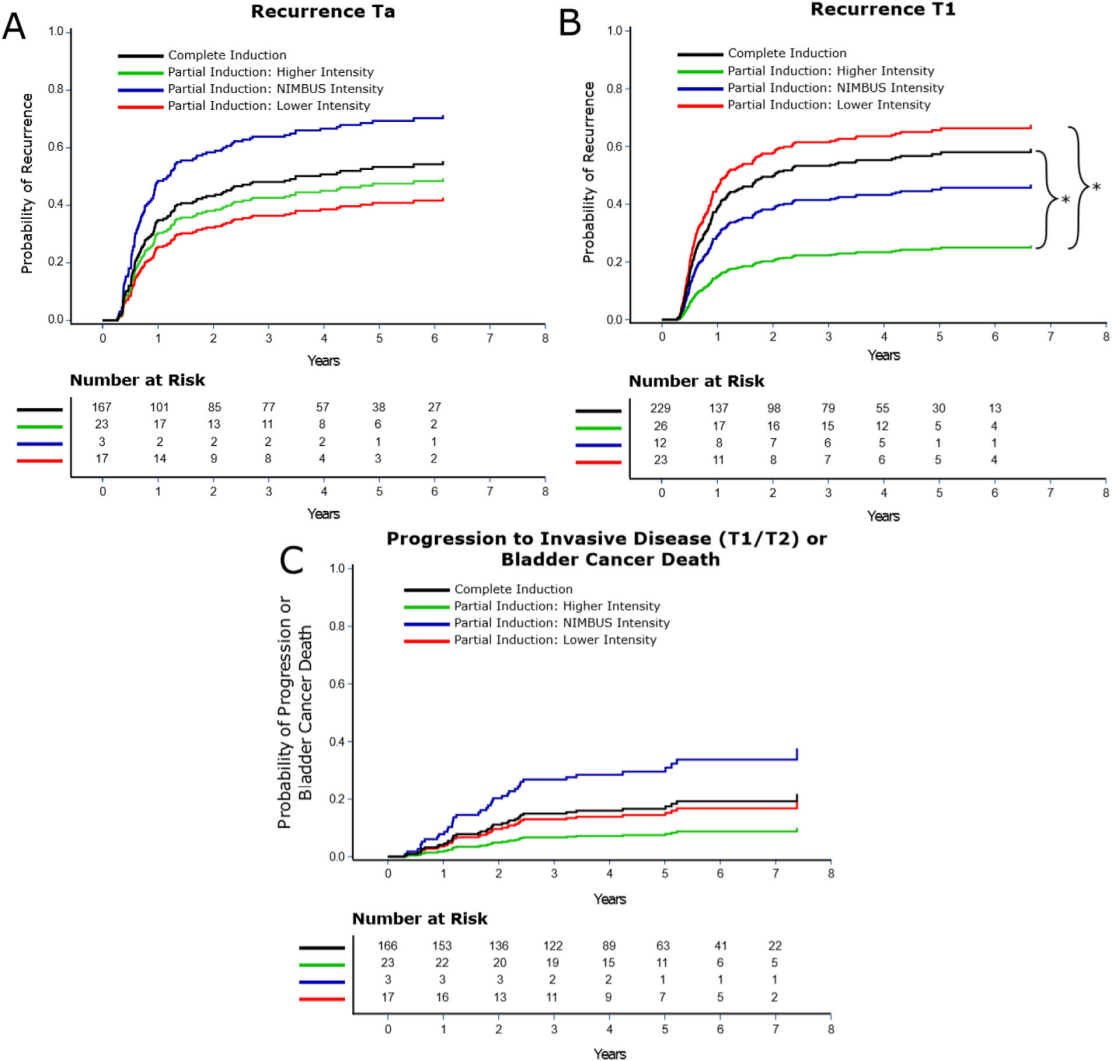
Cumulative incidence plots showing the probability of (A) disease recurrence in HG Ta and (B) T1 disease by partial BCG induction subgroups. Brackets in Figure 4B indicate statistically significant differences between the respective curves based on the adjusted Fine-Gray model (Supplementary Tables 2–10). (C) The probability of progression to invasive disease (T1 or T2) in patients diagnosed with HG Ta by partial BCG induction groups. Data are from Fine and Gray competing risk models adjusted for propensity score. BCG = bacillus Calmette-Guérin; HG = high grade.

Patients who underwent NIMBUS intensity versus complete BCG induction appeared to have a potentially clinically significantly increased risk of disease recurrence if they had HG Ta disease (CIn 69.4% vs 53.3% at 5 yr, *p* =  0.57; Fig. 4A), but not if they had T1 disease (CIn 45.7% vs 58.0% at 5 yr, *p* =  0.43; Fig. 4B), although these differences were not statistically significant. There were no differences in the risk of bladder cancer death among patients with T1 disease (CIn 9.6% vs 11.9% at 5 yr, *p* =  0.81). Bladder cancer death could not be examined for HG Ta disease due to a low number of events. Patients with HG Ta disease who underwent NIMBUS intensity versus complete BCG appeared to have a potentially clinically significantly increased risk of progression to invasive disease or bladder cancer death (CIn 30.9% vs 17.5% at 5 yr, *p* =  0.54; Fig. 4C), although this was not statistically significant.

Our exploratory subgroup was limited by a low sample size, preventing us from drawing any definitive conclusions from the results (Supplementary Fig. 2A–C).

## 4 Discussion

In this national study of veterans with high-risk NMIBC, we found no difference in bladder cancer outcomes by receipt of partial (fewer than five instillations) versus complete (five or more instillations) BCG induction. While not statistically significant and limited by sample size, post hoc analyses revealed potentially clinically relevant increases in the risk of recurrence and progression among HG Ta patients undergoing NIMBUS-intensity partial induction, but not among those undergoing higher-intensity partial induction.

Similar to our study, a recently published phase III clinical trial (NIMBUS) examined the impact of reducing the number of BCG instillations in patients with high-risk NMIBC [10]. In this study, patients were randomly assigned to standard BCG or reduced BCG frequency (BCG weekly at weeks 1, 2, and 6). The trial was stopped prematurely after finding a significantly higher rate of tumor recurrence (27% vs 12%) and progression (3.6% vs 0.6%) among patients who were randomized to reduced BCG frequency [10].

Our findings may differ from those of the NIMBUS trial due to details of the partial BCG induction. The NIMBUS trial introduced a 4-wk gap between induction instillations #2 and #6 in the partial induction arm—substantially reduced treatment intensity compared with weekly instillations of BCG for 6 wk. To compare our partial induction group with NIMBUS, we performed post hoc subgroup analyses categorizing partial BCG induction into higher-intensity, NIMBUS-intensity, and lower-intensity partial induction (Fig. 2). A subset of patients with HG Ta disease who received NIMBUS-intensity partial BCG had substantially increased risks of recurrence and progression. These differences were not statistically significant due to a low number of patients in the NIMBUS-intensity group, but are consistent with the NIMBUS trial as well as with prior research indicating that maximal immune response can be achieved after four weekly instillations of BCG [19], [20]. As such, higher-intensity partial induction, defined as three to four BCG instillations delivered weekly without prolonged gaps between doses, may be more similar to a complete BCG induction course than the NIMBUS-intensity partial BCG.

Contrary to what one would expect, in the HG Ta subgroup, lower-intensity partial induction was associated with the lowest rate of disease recurrence. However, in the subgroup of T1 patients, lower-intensity partial induction was associated with the highest rate of recurrence. The discordance between these two findings may be explained by the low sample sizes in these subgroups as well as unmeasured confounding. Overall, future prospective work with a greater number of patients is needed to confirm our findings. Such research would help determine the most appropriate partial BCG induction pattern benefiting the greatest number of patients during global shortages.

The methodological strengths of our study include a large national sample size, clinically relevant outcome measures, and the use of full-text pathology reports to determine tumor recurrences and progression to invasive bladder cancer. There are also important limitations. First, confounding is a risk for all observational and retrospective studies. We attempted to combat this limitation with propensity score adjustment. However, it is likely that unmeasured patient factors such as adverse reactions led to treatment with partial BCG. Provider preferences and health system factors such as staffing and adherence to societal guidelines could also have affected BCG administration, and it should be noted that our study is from a time prior to national BCG shortages. However, unobserved confounding would have to be substantial for us to miss true worsening in cancer outcomes, which was indicated by E-values of >1.6 [21]. Second, the generalizability of our results may be limited. Our study population consisted of VA patients older than 65 yr; these patients have more comorbidities and lower socioeconomic status than the general population [22]. However, men older than 65 yr comprise the most common group diagnosed with bladder cancer [23]. Third, only 28.7 % of veterans with high-risk NMIBC received BCG and were included in our study, which may be concerning for a selection bias. BCG utilization has increased substantially in accordance with American Urological Association (AUA) guidelines over the last 20 yr [24]. A small regional study including veterans diagnosed in 2016–2017 found that 45/68 high-risk patients received BCG [25]. However, a large population-based study using SEER-Medicare data found that only 13–20% of patients with high-risk NMIBC received guideline-recommended intravesical BCG therapy between 1992 and 2002 [26]. The low percentage of veterans receiving BCG in our study may be explained by historically low compliance to AUA guidelines, regional variation in care, gaps in healthcare access and patient education, and potentially not all facilities forwarding pharmacy data to the CDW. However, as veterans usually get treatment in one facility, it is unlikely that these issues led to misclassification of complete versus partial BCG. Finally, differential follow-up could have affected our results. However, we found no significant differences in the median number of surveillance cystoscopies per year between patients who underwent partial and those who underwent complete BCG induction (median 2.6 vs 2.9, *p* =  0.29 from equality of median test).

In spite of these limitations, our study has important implications. First, our data suggest that some partial BCG induction courses that are similarly effective to standard 6-wk induction BCG may exist, thus providing an added perspective to the NIMBUS study. Second, the NIMBUS study ended early and thus could not adequately assess progression to bladder cancer death. In fact, all death events (2.9%) were non–bladder cancer deaths [10]. Our results add to the evidence as we found no difference in bladder cancer death by partial versus complete BCG induction, with a median length of follow-up of 4.5 yr. Third, recent global shortages of BCG secondary to increased global demand [27] and manufacturing issues [9] have resulted in medication rationing, incomplete therapy regimens, and treatment delays [8], [9], [27]. Our findings suggest that future research examining different intravesical therapy administration patterns may help determine how to optimize BCG delivery for the greatest number of patients, especially during global shortages.

## 5 Conclusions

Patients with high-risk NMIBC who underwent partial BCG induction experienced outcomes similar to those who received complete BCG induction. Given the observational and retrospective nature of this study, our findings are subject to unmeasured confounding and should be interpreted with caution. We do not advocate for reducing widely adopted intravesical BCG therapy practices based on our findings. Instead, our findings provide a rationale for additional prospective trials to determine how to best maximize the clinical benefits of BCG for the greatest number of patients during global shortages.

  ***Author contributions*:** Michael E. Rezaee had full access to all the data in the study and takes responsibility for the integrity of the data and the accuracy of the data analysis.

  *Study concept and design*: Rezaee, Schroeck, Ismail.

*Acquisition of data*: Lynch, Schroeck.

*Analysis and interpretation of data*: Ismail, Schroeck, Rezaee, Lynch.

*Drafting of the manuscript*: Rezaee, Schroeck, Okorie.

*Critical revision of the manuscript for important intellectual content*: Rezaee, Schroeck, Okorie, Seigne, Ismail, Lynch.

*Statistical analysis*: Ismail, Schroeck.

*Obtaining funding*: Schroeck.

*Administrative, technical, or material support*: Ismail.

*Supervision*: Lynch, Schroeck, Seigne.

*Other*: None.

  ***Financial disclosures:*** Michael E. Rezaee certifies that all conflicts of interest, including specific financial interests and relationships and affiliations relevant to the subject matter or materials discussed in the manuscript (eg, employment/affiliation, grants or funding, consultancies, honoraria, stock ownership or options, expert testimony, royalties, or patents filed, received, or pending), are the following: None.

  ***Funding/Support and role of the sponsor*:** This study was supported using resources and facilities at the White River Junction Department of Veterans Affairs (VA) Medical Center, the VA Salt Lake City Health Care System, and the VA Informatics and Computing Infrastructure (VINCI; VA HSR RES 13-457). VA and Centers for Medicare & Medicaid Services data are provided with support from the VA Information Resource Center (project numbers SDR 02-237 and 98-004). Florian R. Schroeck is supported by Health Services Research and Development grant I01HX002780 from the Department of Veterans Affairs. Opinions expressed in this manuscript are those of the authors and do not constitute official positions of the U.S. Federal Government or the Department of Veterans Affairs.
