# Supplementary material for: Partial Versus Complete Bacillus Calmette-Guérin Intravesical Therapy and Bladder Cancer Outcomes in High-risk Non–muscle-invasive Bladder Cancer: Is NIMBUS the Full Story?
Source: Eur Urol Open Sci. 2021 Feb 16;26:35–43. doi: 10.1016/j.euros.2021.01.009 (PMC8317819; doi:10.1016/j.euros.2021.01.009)

**Supplementary Figure 2A–C**: Cumulative incidence plots showing the probability of disease recurrence (Panel A) and bladder cancer death (Panel B) by HG Ta versus T1 disease and by complete vs. exploratory BCG. Panel C shows the probability of progression to invasive disease (T1 or T2) in patients diagnosed with HG Ta by complete vs. exploratory BCG. Data are from Fine and Gray ompeting risk models adjusted for propensity score.


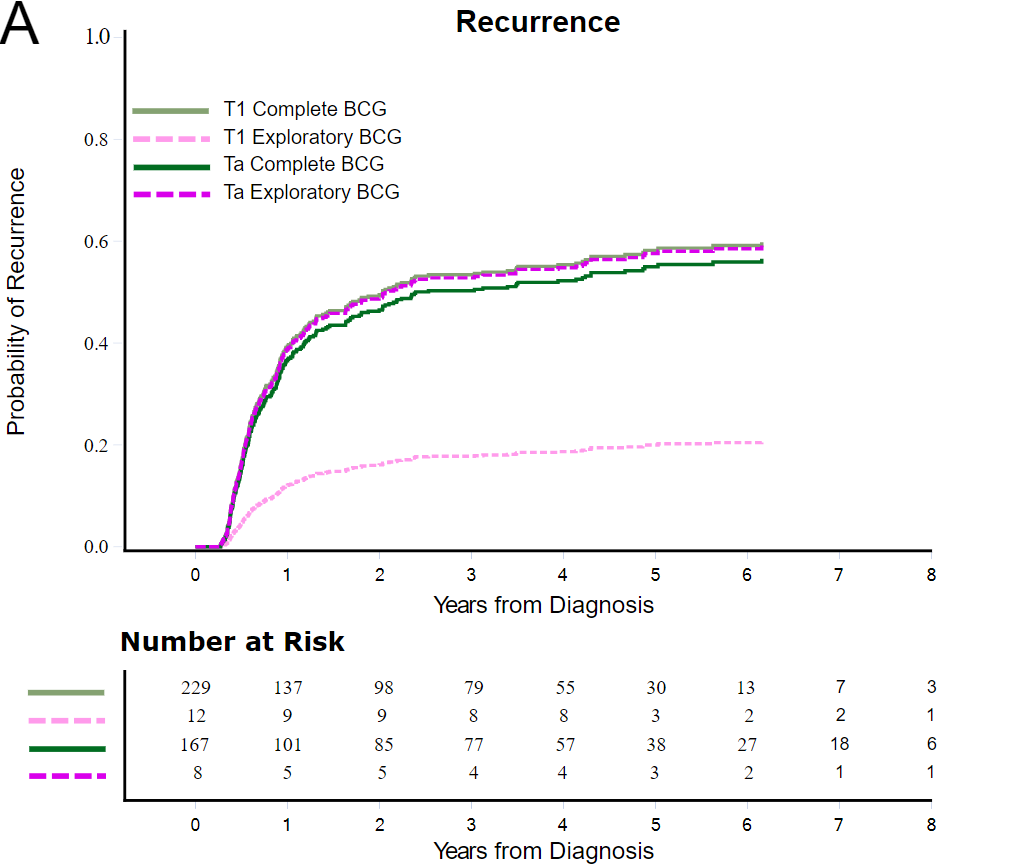


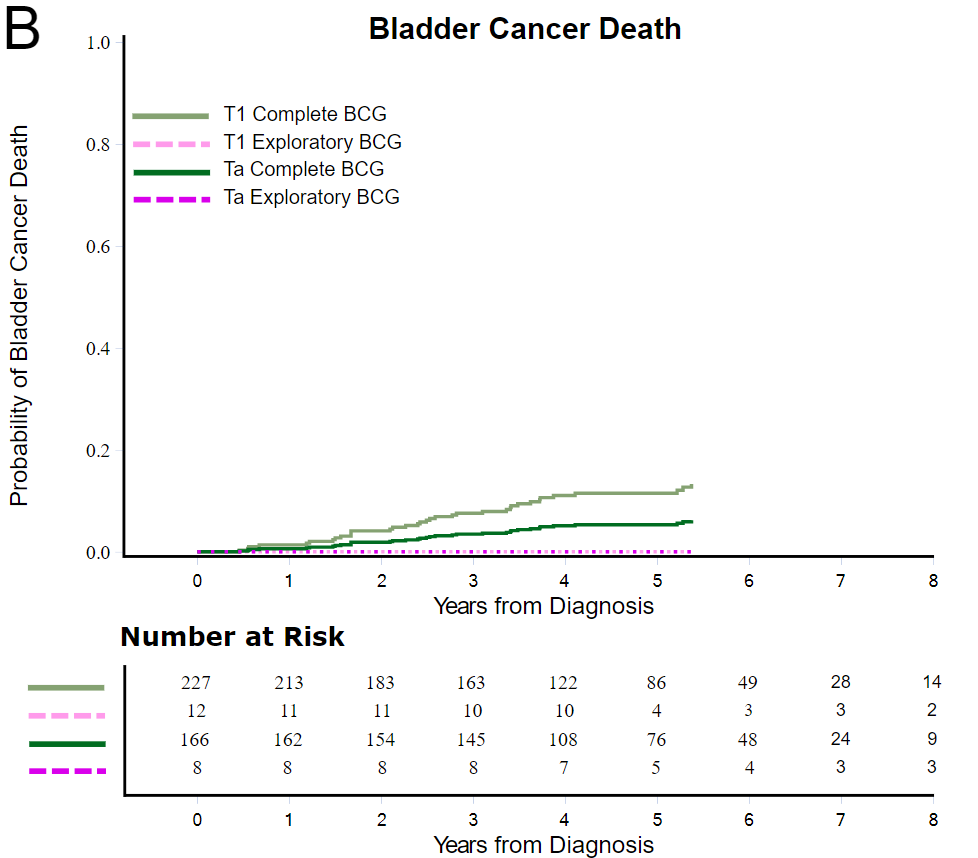


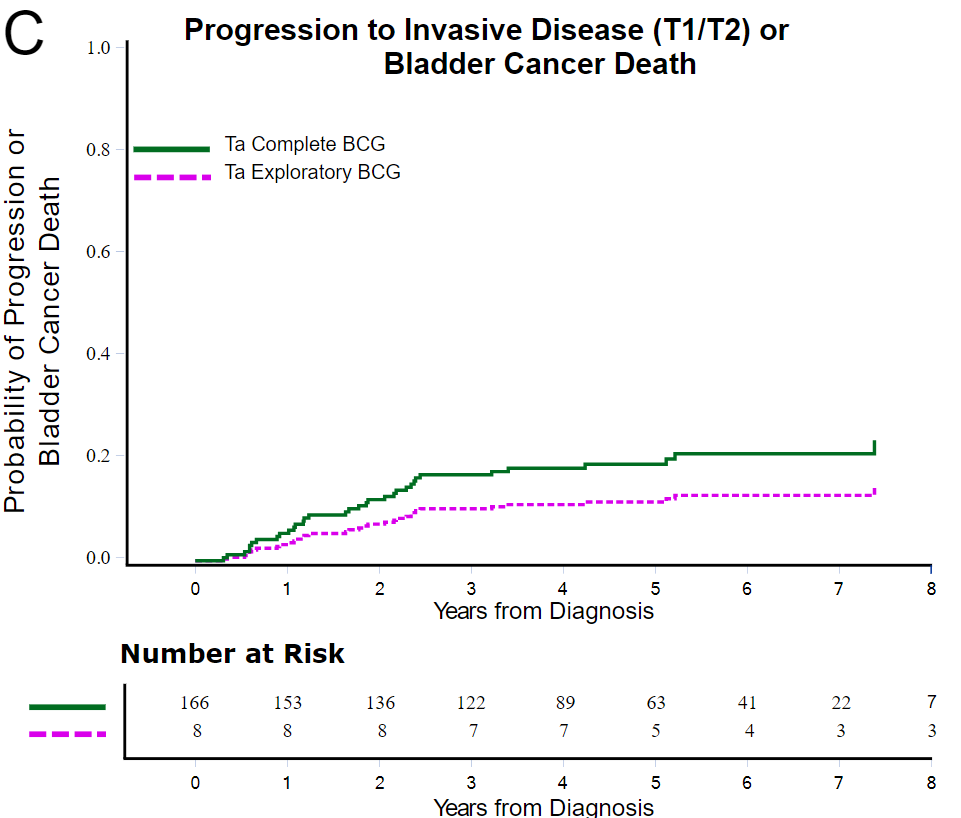

Supplement: Supplementary file 1 [file mmc1.docx]
