# Supplementary material for: Partial Versus Complete Bacillus Calmette-Guérin Intravesical Therapy and Bladder Cancer Outcomes in High-risk Non–muscle-invasive Bladder Cancer: Is NIMBUS the Full Story?
Source: Eur Urol Open Sci. 2021 Feb 16;26:35–43. doi: 10.1016/j.euros.2021.01.009 (PMC8317819; doi:10.1016/j.euros.2021.01.009)

**Supplementary material**

***Supplemental Methods and Results***

***Details on Fine-Gray model construction***

**Methods:**

First, propensity score adjusted Fine-Gray competing risks regression was used to evaluate the association of partial BCG induction with risk of disease recurrence. In this model, death was modeled as a competing risk. The follow-up period started with the date of diagnosis and ended with the date of death, last contact with the VA system, or at the end of the study (December 31, 2014), whichever came first. Patients were censored at last contact with the VA system or at the end of the study. Second, a similar propensity score adjusted Fine-Gray model was fitted to assess the association of partial BCG induction with bladder cancer death. In this model, death from causes other than bladder cancer was modeled as the competing risk. These analyses were stratified by Ta (HG or associated with CIS) versus T1 disease at the time of diagnosis. Third, a Fine-Gray regression model was used to assess the risk of progression to invasive bladder cancer (T1 or T2) or bladder cancer death among patients initially diagnosed with HG Ta disease. Death from causes other than bladder cancer was modeled as the competing risk for this model.

***Patients who did versus did not undergo BCG Maintenance & Outcomes***

Methods:

We *a priori* categorized patients into those who did undergo complete BCG maintenance (at least two doses) versus those that did not (0 or 1 doses) in line with the International Bladder Cancer Group definitions [1]. The average number of BCG instillations in the complete BCG maintenance group was 3.1 versus 0.1 among those that did not undergo complete BCG maintenance. In fact, only 15 patients (5.1%) in the group that did not undergo complete maintenance had 1 dose of maintenance BCG administered; all others underwent no maintenance instillations. Thus, we will refer to these groups as complete vs. no maintenance in the results. We again used propensity score adjusted Fine-Gray competing risk regression models to assess associations between maintenance BCG and outcomes.

**Results:**

Among the 426 patients who underwent complete BCG induction, 110 (25.8%) underwent complete BCG maintenance while 316 (74.2%) did not. Baseline patient characteristics did not differ significantly by BCG maintenance status (Supplementary Table 1).

No vs. complete BCG maintenance was not statistically significantly associated with increased risk of disease recurrence for patients diagnosed with HG Ta (CIn 55.7% vs. 48.5% at 5 years, p=0.42) or T1 (CIn 60.1% vs. 48.9% at 5 years, p=0.15) disease (Supplementary Figure 1A). Similarly, no vs. complete BCG induction was not statistically significantly associated with increased risk of bladder cancer death for patients diagnosed with HG Ta (CIn 6.4% vs. 2.4% at 5 years, p=0.35) or T1 (CIn 12.1% vs. 9.4% at 5 years, p=0.59) disease (Supplementary Figure 1B). Among patients who received no maintenance BCG, there was no significantly increased risk of progression to invasive disease or bladder cancer death when diagnosed with HG Ta (CIn 20.1 % vs. 18.0% at 5 years, p=0.77, Supplementary Figure 1C).

Comments:

The oncologic benefit of a 3-week BCG maintenance regimen in addition to BCG induction is well established [2]. We did not observe statistically significant differences in study outcomes by receipt of BCG maintenance. However, point estimates indicated a likely clinically relevant association of no BCG maintenance with poorer outcomes. Failure to achieve statistical significance is likely explained by smaller sample sizes among our subgroups. The implications of reducing BCG instillations below the standard 3-week maintenance regimen are unknown and could not be assessed because only 15 patients received a single maintenance dose.

**References**

1. Kamat AM, Sylvester RJ, Böhle A, et al. Definitions, End Points, and Clinical Trial Designs for Non-Muscle-Invasive Bladder Cancer: Recommendations From the International Bladder Cancer Group. *J Clin Oncol.* 2016;34(16):1935-1944.
2. Kamat AM, Flaig TW, Grossman HB, et al. Consensus statement on best practice management regarding the use of intravesical immunotherapy with BCG for bladder cancer. *Nature Reviews Urology.* 2015;12(4):225-235.

**Supplementary Table 1:** **Patient Characteristics Among those who Received Complete BCG induction by No and Complete BCG Maintenance**

| **Category** | **Overall:**  **(n= 426)** | **Complete Induction: Complete Maintenance (n= 110 )** | **Complete Induction: No**  **Maintenance (n= 316 )** | **P_value*** |
| --- | --- | --- | --- | --- |
| Age, mean (SD), y | 76 (6.7) | 75 (6.5) | 77 (6.7) | 0.07 |
| Male Sex,** N (%) | >415 (>97.4%) | >99 (>90.0%) | >305 (>96.5%) | 0.94 |
| Race, N (%) |  |  |  | 0.97 |
| White | 358 (84%) | 92 (83.6%) | 266 (84.2%) |  |
| Black** | 24 (5.6%) | < 11 | 18 (5.7%) |  |
| Asian** | < 11 | < 11 | < 11 |  |
| Hispanic** | < 11 | < 11 | < 11 |  |
| Native American** | < 11 | < 11 | < 11 |  |
| Unknown** | 32 (7.5%) | < 11 | >30 (>7.0%) |  |
| Comorbidity, N (%) |  |  |  | 0.38 |
| 0 | 59 (13.8%) | 19 (17.3%) | 40 (12.7%) |  |
| 1 | 110 (25.8%) | 29 (26.4%) | 81 (25.6%) |  |
| 2 | 110 (25.8%) | 26 (23.6%) | 84 (26.6%) |  |
| >=3 | 147 (34.5%) | 36 (32.7%) | 111 (35.1%) |  |
| Year of diagnosis,** N (%) |  |  |  | 0.19 |
| 2005 | < 11 | < 11 | < 11 |  |
| 2006 | 57 (13.4%) | < 11 | 47 (14.9%) |  |
| 2007 | 62 (14.6%) | 14 (12.7%) | 48 (15.2%) |  |
| 2008 | 73 (17.1%) | 12 (10.9%) | 61 (19.3%) |  |
| 2009 | 73 (17.1%) | 27 (24.5%) | 46 (14.6%) |  |
| 2010 | >70 (18.0%) | 26 (23.6%) | >50 (16.0%) |  |
| 2011 | >70 (17.0%) | 18 (16.4%) | >50 (17.0%) |  |
| Proportion living in ZIP code with >=25% college graduates, N (%) | 196 (46%) | 49 (44.5%) | 147 (46.5%) | 0.71 |
| Living in urban vs. rural area, N (%) |  |  |  | 0.81 |
| Urban | 250 (58.7%) | 62 (56.4%) | 188 (59.5%) |  |
| Stage, N (%)* |  |  |  | 0.93 |
| Ta (high grade or with CIS) | 167 (39.2%) | >38 (>34.5%) | 126 (39.9%) |  |
| T1 | 229 (53.8%) | 60 (54.5%) | 169 (53.5%) |  |
| Carcinoma in situ only** | 30 (7%) | < 11 | 21 (6.6%) |  |
| Carcinoma in situ, N (%) | 125 (29.3%) | 33 (30%) | 92 (29.1%) | 0.98 |
| Bladder Cancer Grade, N (%) |  |  |  | 0.19 |
| High | 398 (93.4%) | >90 (> 89%) | 299 (94.6%) |  |
| Low (all T1 tumors) | 28 (6.6%) | >9 (>9%) | 17 (5.4%) |  |
| * From Chi-square test for categorical variable and Wilcoxon test for continuous variables whose median and IQR were presented. Missing observations were excluded for analysis.  ** Exact numbers not shown to protect confidentiality. | | | | |

**Supplementary Figure 1 A-C**: Cumulative incidence plots showing the probability of disease recurrence (Panel A) and bladder cancer death (Panel B) by HG Ta versus T1 disease among patients with complete BCG induction by BCG maintenance status. Panel C shows the probability of progression to invasive disease (T1 or T2) in patients diagnosed with HG Ta with complete BCG induction by BCG maintenance status. Data are from Fine and Gray competing risk models adjusted for propensity score. No statistically significant differernces were observed beween groups.


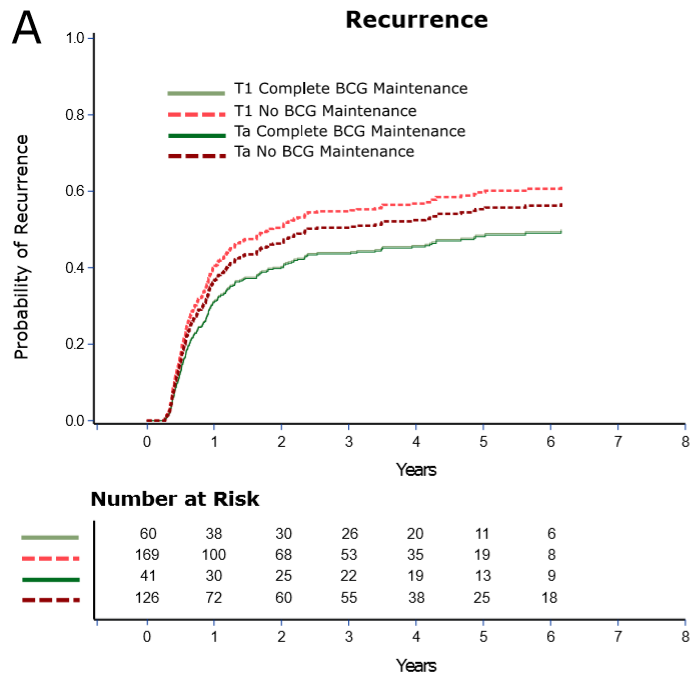


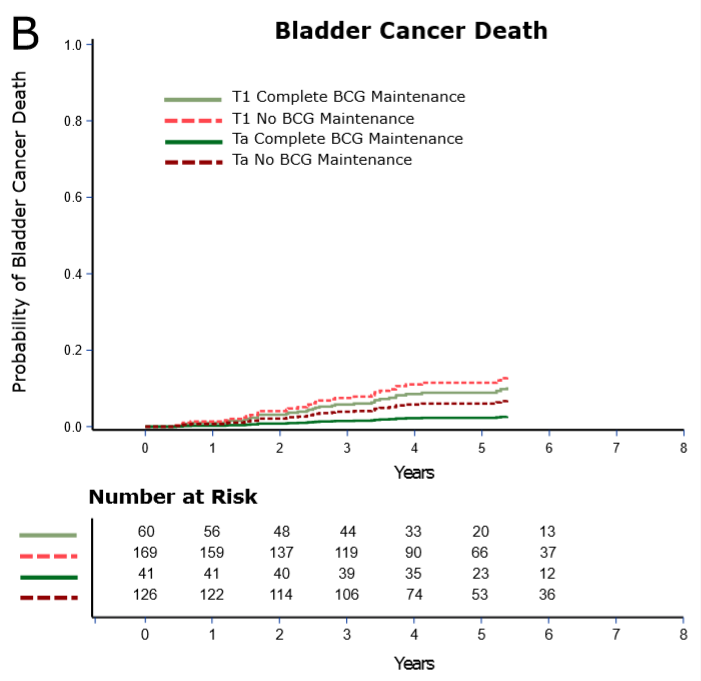


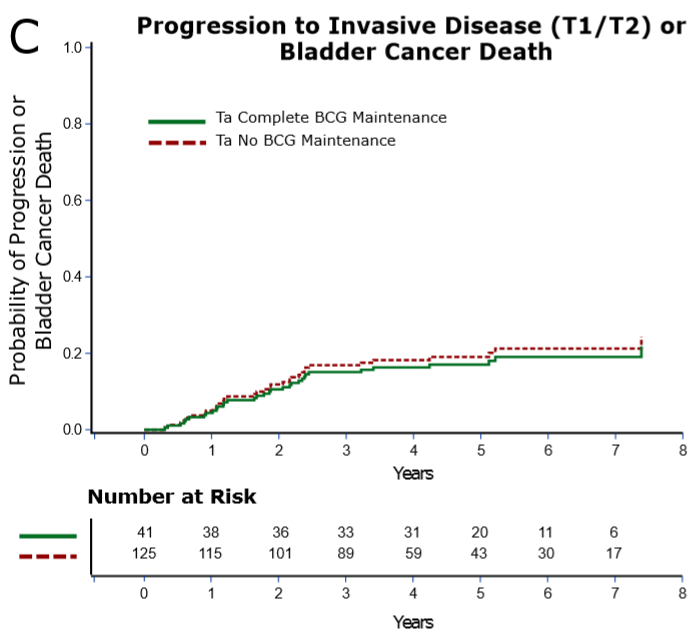

Supplement: Supplementary file 2 [file mmc2.docx]
