# Supplementary material for: Partial Versus Complete Bacillus Calmette-Guérin Intravesical Therapy and Bladder Cancer Outcomes in High-risk Non–muscle-invasive Bladder Cancer: Is NIMBUS the Full Story?
Source: Eur Urol Open Sci. 2021 Feb 16;26:35–43. doi: 10.1016/j.euros.2021.01.009 (PMC8317819; doi:10.1016/j.euros.2021.01.009)
Supplement: Supplementary file 3 [file mmc3.docx]

***Detailed model output from the Fine-Gray regression models***

Supplementary Table 2: Fine-Gray regression model assessing the association between partial vs. complete BCG induction and recurrence among patients with HG Ta disease.

| **Parameter** | **P value** | **Hazard Ratio** | **CI U** | **CI L** |
| --- | --- | --- | --- | --- |
| Complete Induction T1 | 0.5666 | 1.082 | 0.827 | 1.416 |
| Partial Induction T1 | 0.3632 | 0.823 | 0.541 | 1.252 |
| Partial Induction Ta | 0.3848 | 0.81 | 0.503 | 1.303 |
| Propensity score | 0.155 | 1.043 | 0.984 | 1.106 |

Supplementary Table 3: Fine-Gray regression model assessing the association between partial vs. complete BCG induction and recurrence among patients with T1 disease.

| **Parameter** | **P value** | **Hazard Ratio** | **CI U** | **CI L** |
| --- | --- | --- | --- | --- |
| Partial Induction T1 | 0.1885 | 0.761 | 0.506 | 1.143 |
| Complete Induction Ta | 0.5666 | 0.924 | 0.706 | 1.21 |
| Partial Induction Ta | 0.222 | 0.748 | 0.47 | 1.192 |
| Propensity Score | 0.155 | 1.043 | 0.984 | 1.106 |

Supplementary Table 4: Fine-Gray regression model assessing the association between partial vs. complete BCG induction and bladder cancer death among patients with HG Ta disease.

| **Parameter** | **P value** | **Hazard Ratio** | **CI U** | **CI L** |
| --- | --- | --- | --- | --- |
| Complete Induction T1 | 0.0411 | 2.187 | 1.032 | 4.634 |
| Partial Induction T1 | 0.2139 | 1.917 | 0.687 | 5.349 |
| Partial Induction Ta | 0.8651 | 0.875 | 0.188 | 4.07 |
| Propensity Score | 0.4 | 1.053 | 0.933 | 1.189 |

Supplementary Table 5: Fine-Gray regression model assessing the association between partial vs. complete BCG induction and bladder cancer death among patients with T1 disease.

| **Parameter** | **P value** | **Hazard Ratio** | **CI U** | **CI L** |
| --- | --- | --- | --- | --- |
| Partial Induction T1 | 0.7728 | 0.877 | 0.359 | 2.143 |
| Complete Induction Ta | 0.0411 | 0.457 | 0.216 | 0.969 |
| Partial Induction Ta | 0.2156 | 0.4 | 0.094 | 1.705 |
| Propensity Score | 0.4 | 1.053 | 0.933 | 1.189 |

Supplementary Table 6: Fine-Gray regression model assessing the association between partial vs. complete BCG induction and progression or bladder cancer death among patients with HG Ta disease.

| **Parameter** | **P value** | **Hazard Ratio** | **CI U** | **CI L** |
| --- | --- | --- | --- | --- |
| Partial Induction group | 0.3657 | 0.667 | 0.277 | 1.605 |
| Propensity Score | 0.6864 | 0.959 | 0.783 | 1.175 |

Supplementary Table 7: Fine-Gray regression model assessing the association between partial BCG induction subgroups vs. complete BCG induction and recurrence among patients with HG Ta disease.

| **Parameter** | **P value** | **Hazard Ratio** | **CI U** | **CI L** |
| --- | --- | --- | --- | --- |
| Partial Induction: Low Intensity | 0.3422 | 0.689 | 0.319 | 1.487 |
| Partial Induction: NIMBUS Intensity | 0.5727 | 1.551 | 0.337 | 7.136 |
| Partial Induction: High Intensity | 0.5864 | 0.846 | 0.463 | 1.546 |
| Propensity score NIMBUS intensity | 0.633 | 1.008 | 0.975 | 1.043 |
| Propensity score higher intensity partial BCG | 0.6679 | 1.026 | 0.911 | 1.156 |
| Propensity score complete induction | 0.3336 | 1.067 | 0.935 | 1.218 |

Supplementary Table 8: Fine-Gray regression model assessing the association between partial BCG induction subgroups vs. complete BCG induction and recurrence among patients with T1 disease.

| **Parameter** | **P value** | **Hazard Ratio** | **CI U** | **CI L** |
| --- | --- | --- | --- | --- |
| Partial Induction: Low Intensity | 0.3769 | 1.254 | 0.759 | 2.072 |
| Partial Induction: NIMBUS Intensity | 0.4355 | 0.704 | 0.291 | 1.702 |
| Partial Induction: High Intensity | 0.0248 | 0.331 | 0.126 | 0.869 |
| Propensity score NIMBUS intensity | 0.1035 | 0.983 | 0.963 | 1.004 |
| Propensity score higher intensity partial BCG | 0.3402 | 1.043 | 0.957 | 1.137 |
| Propensity score complete induction | 0.5833 | 0.971 | 0.874 | 1.079 |

Supplementary Table 9: Fine-Gray regression model assessing the association between partial BCG induction subgroups vs. complete BCG induction and bladder cancer death among patients with T1 disease.

| **Parameter** | **P value** | **Hazard Ratio** | **CI U** | **CI L** |
| --- | --- | --- | --- | --- |
| Partial Induction: Low Intensity | 0.4637 | 1.527 | 0.492 | 4.741 |
| Partial Induction: NIMBUS Intensity | 0.8135 | 0.792 | 0.114 | 5.492 |
| Partial Induction: High Intensity | 0.282 | 0.321 | 0.041 | 2.544 |
| Propensity score NIMBUS intensity | 0.2371 | 0.972 | 0.927 | 1.019 |
| Propensity score higher intensity partial BCG | 0.8433 | 0.978 | 0.788 | 1.214 |
| Propensity score complete induction | 0.6956 | 1.05 | 0.823 | 1.34 |

Supplementary Table 10: Fine-Gray regression model assessing the association between partial BCG induction subgroups vs. complete BCG induction and progression or bladder cancer death among patients with HG Ta disease.

| **Parameter** | **P value** | **Hazard Ratio** | **CI U** | **CI L** |
| --- | --- | --- | --- | --- |
| Partial Induction: Low Intensity | 0.7998 | 0.858 | 0.264 | 2.795 |
| Partial Induction: NIMBUS Intensity | 0.5407 | 1.925 | 0.236 | 15.681 |
| Partial Induction: High Intensity | 0.2538 | 0.428 | 0.1 | 1.839 |
| Propensity score NIMBUS intensity | 0.2751 | 1.038 | 0.971 | 1.109 |
| Propensity score higher intensity partial BCG | 0.0378 | 1.142 | 1.007 | 1.294 |
| Propensity score complete induction | 0.2003 | 0.844 | 0.652 | 1.094 |
